# Neural Differentiation of Human Adipose Tissue-Derived Stem Cells Involves Activation of the Wnt5a/JNK Signalling

**DOI:** 10.1155/2015/178618

**Published:** 2015-05-27

**Authors:** Sujeong Jang, Jong-Seong Park, Han-Seong Jeong

**Affiliations:** Department of Physiology, Chonnam National University Medical School, Gwangju 501746, Republic of Korea

## Abstract

Stem cells are a powerful resource for cell-based transplantation therapies, but understanding of stem cell differentiation at the molecular level is not clear yet. We hypothesized that the Wnt pathway controls stem cell maintenance and neural differentiation. We have characterized the transcriptional expression of Wnt during the neural differentiation of hADSCs. After neural induction, the expressions of Wnt2, Wnt4, and Wnt11 were decreased, but the expression of Wnt5a was increased compared with primary hADSCs in RT-PCR analysis. In addition, the expression levels of most Fzds and LRP5/6 ligand were decreased, but not Fzd3 and Fzd5. Furthermore, Dvl1 and RYK expression levels were downregulated in NI-hADSCs. There were no changes in the expression of ß-catenin and GSK3ß. Interestingly, Wnt5a expression was highly increased in NI-hADSCs by real time RT-PCR analysis and western blot. Wnt5a level was upregulated after neural differentiation and Wnt3, Dvl2, and Naked1 levels were downregulated. Finally, we found that the JNK expression was increased after neural induction and ERK level was decreased. Thus, this study shows for the first time how a single Wnt5a ligand can activate the neural differentiation pathway through the activation of Wnt5a/JNK pathway by binding Fzd3 and Fzd5 and directing Axin/GSK-3ß in hADSCs.

## 1. Background

Mesenchymal stem cells (MSCs) reside in the bone marrow, peripheral blood, and adipose tissue and have a therapeutic potential in diseases such as multiple sclerosis [1], diabetes [2], stroke [3, 4], and neurodegenerative disease [5]. MSCs self-renew and differentiate into bone, fat tissue, cartilage, muscle, marrow stroma, tendon, and ligament, both* in vivo* and* in vitro*, under appropriate culture conditions [6, 7]. Understanding differentiation is important for several reasons. Firstly, differentiation is based on the process of acquiring specific functions so that stem cell research is likely to focus on improving the ability to guide the differentiation and to control their survival and proliferation [8, 9]. Secondly, understanding differentiation may provide a proper strategy for clinical treatment of stem cells in neurological disorders. For therapeutic applications of MSCs, how stem cells switch from proliferation to differentiation and consistently sustain their abilities is very important. Recently, Wnt signalling has been implicated in the control of MSC differentiation [10, 11], including osteogenic [12, 13], chondrogenic, adipogenic, and myogenic differentiation [14].

Wnts are a family of glycoproteins that can signal through distinct intracellular pathways and regulate many developmental events in vertebrates and mammals [13, 15]. As many as 19 mammalian Wnt homologues are known and shared features of all Wnts include a signal sequence for secretion, several highly charged amino acid residues, and many glycosylation sites [16]. Wnt proteins signal through two different pathways by binding with Frizzled (Fzd) receptors, which are seven-pass transmembrane proteins and are found as first receptors to transduce a Wnt signal [17]. At the cytoplasmic side, Fzd may interact directly with the Dishevelled (Dvl) protein, a known mediator of Wnt signalling [16]. In canonical pathway, Wnt/Fzd complex signals through activation of Dvl to inhibit the degradation of *β*-catenin by the glycogen synthase kinase-3*β* (GSK3*β*). The accumulation of the stabilized *β*-catenin in cytoplasm is increased and enters the nucleus where it induces transcription of target gene expression. Noncanonical pathway, which is independent of *β*-catenin, mediates several cellular processes through monomeric GTPases of the Rho family, mitogen-activated protein kinase like Jun N-terminal kinase (JNK), and changes in intracellular calcium levels [10, 11, 16–20].

The Wnt signalling pathway plays an important role in the development of the central nervous system and regulates the formation and function of neuronal circuits by controlling neuronal differentiation [21]. Several groups reported that Wnt3a and Wnt5a increased neural differentiation of human or mouse neuronal progenitor cells [15] and Wnt3a promoted the neuronal differentiation of rat MSCs by upregulation of the *β*-catenin expression [11]. Wnts also stimulated neural differentiation from embryonic, somatic, and neural stem cells [22, 23]. However, the Wnt pathway in neural differentiation of human adipose tissue-derived stem cells (hADSCs) is not studied yet. In the present study, we established neural induced-hADSCs (NI-hADSCs) following our previous study [9] and figured out the Wnt signalling and related pathways in NI-hADSCs compared with primary hADSCs.

## 2. Materials and Methods

### 2.1. Preparation of hADSCs and Neural Induction of hADSCs

In this study, we used human ADSCs, which were already isolated and characterized in our previous study [9]. In brief, fat tissue from the human earlobe was obtained from ten healthy donors 4–20 years of age according to the Guidelines of the Ethics Committee at Chonnam National University Medical School (IRB:I-2009-03-016). Isolated hADSCs were maintained in Dulbecco's modified Eagle's medium (DMEM, Hyclone, Logan, UT, USA) supplemented with 10% fetal bovine serum (FBS, Hyclone) and 1% penicillin-streptomycin (Hyclone) at 37°C humidified incubator with 5% CO_2_. The hADSCs used in the present study were from passages 2 to 5. To induce neural differentiation, hADSCs were grown in DMEM containing 1% FBS and supplementary 100 ng/mL basic fibroblast growth factor (bFGF, Invitrogen Co., Carlsbad, CA, USA) for seven days and were then incubated in 10 *μ*M forskolin (Sigma Chemical Co., St. Louis, MO, USA) for the next seven days. After that, the cells were subjected to RT-PCR analyses, real time RT-PCR assay, and western blot study.

### 2.2. Reverse Transcriptase-Polymerase Chain Reaction (RT-PCR)

To analyse the relative expression of different mRNAs, the amount of cDNA was normalized based on signals from the ubiquitously expressed GAPDH [9, 24]. Total RNA was extracted from cultured cells by using TRI Reagent (Molecular Research Center Inc., Cincinnati, OH, USA) and reverse-transcribed to cDNA using Reverse Transcription System (Takara Bio Inc., Shiga, Japan). PCR primer (Bioneer Co., Daejeon, Korea) pairs for human were selected to discriminate between cDNA and genomic DNA by using individual primers specific to different exons, when possible. Amplification was carried out at 94°C for 2 min, followed by 35 cycles at 94°C for 1 min, at appropriate annealing temperatures for each primer for 1 min, and at 72°C for 1 min [9]. Forward and reverse PCR oligonucleotide primers selected to amplify the cDNA are listed in Table 1 and RT-PCR products were separated electrophoretically on 2% agarose gels (Sigma Chemical Co.).

### 2.3. Real Time RT-PCR

For real time RT-PCR analyses, cDNA was synthesized using total RNA which was previously prepared from primary hADSCs and NI-hADSCs using TRIzol protocol. Real time RT-PCR was performed with SYBR Green Premix Ex Taq (Takara Bio Inc.) and Thermal Cycler Dice Real Time System (Takara Bio Inc.). Each sample was analysed in four replicate reactions of 20 *μ*L.

### 2.4. Western Blotting

The cells were cultured and Wnt signalling pathway was observed by western blot analysis. Briefly, the cells were washed twice in phosphate-buffered saline (AMRESCO, Solon Ind., Solon, OH, USA) and lysed in a lysis buffer including protease inhibitors and phosphatase inhibitors. Protein concentration in cell lysates was determined by Quant-iT Assay Kit (Molecular Probes, Eugene, OR, USA). An equal amount (15 mg) of protein for each sample was resolved on 10% sodium dodecyl sulphate-polyacrylamide gel electrophoresis and transferred to nitrocellulose membranes (Amersham Pharmacia Biotech, Buckinghamshire, UK). After blocking with 1% skim milk (Sigma Chemical Co.) in PBS-T, the membranes were incubated with specific antibodies for Wnt3a (Cell Signaling Technology, Inc., Danvers, MA, USA, 1 : 1,000), Wnt5a/b (Cell Signaling Technology, 1 : 4,000), Dvl2 (Cell Signaling Technology, 1 : 1,000), Naked1 (Cell Signaling Technology, 1 : 1,000), Axin 1 (Cell Signaling Technology, 1 : 2,000), phosphor-JNK (Santa Cruz Biotechnology, Santa Cruz, CA, USA, 1 : 4,000), JNK (Santa Cruz Biotechnology, 1 : 4,000), phosphor-ERK1/2 (Santa Cruz Biotechnology, 1 : 1,000), ERK1/2 (Santa Cruz Biotechnology, 1 : 4,000), phosphor-PKC (Santa Cruz Biotechnology, 1 : 4,000), PKC (Santa Cruz Biotechnology, 1 : 1,000), phosphor-GSK3*β* (Cell Signaling Technology, 1 : 3,000), GSK3*β* (Cell Signaling Technology, 1 : 3,000), *β*-catenin (Cell Signaling Technology, 1 : 4,000), or GAPDH (Santa Cruz Biotechnology, 1 : 4,000). Following several washes, membranes were subsequently incubated with horseradish peroxidase-conjugated goat anti-rabbit IgG antibody (Cell Signaling Technology). The signals were detected by enhanced chemiluminescence reagents (Santa Cruz Biotechnology) and the density was quantified using ImageJ software [24].

### 2.5. Statistics

Protein and mRNA levels were quantified by measuring the optical density of each band using computer-assisted densitometry (NIH Image analysis program, version 1.61). All values were expressed as the mean ± SEM. The one-way ANOVA test (Bonferroni* post hoc* comparison) was used to analyse differences between groups, with *P* < 0.05 being considered significant.

## 3. Results

### 3.1. Analysis of Wnt Signal-Related Genes in NI-hADSCs

**Figure 1:**
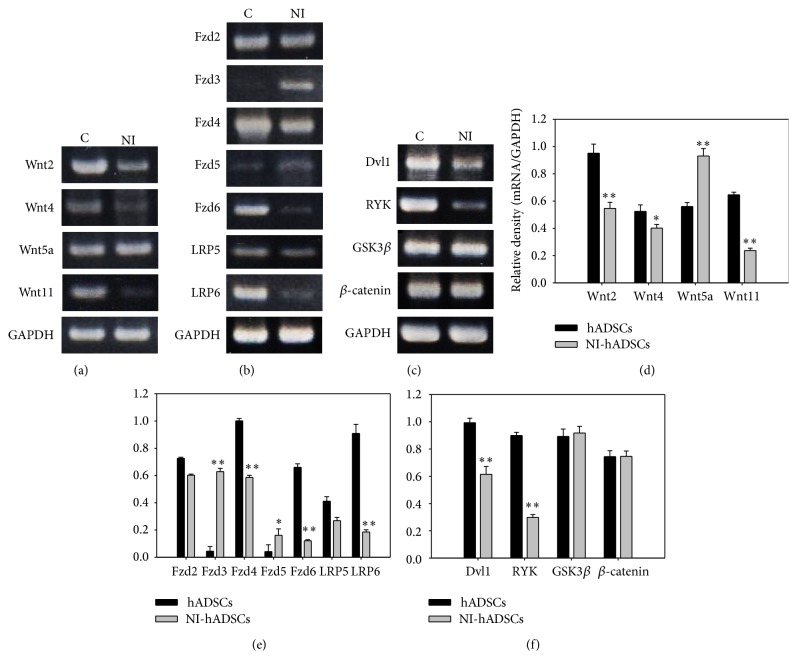
RT-PCR analysis of Wnt pathway-related genes. We demonstrated Wnt genes and Wnt pathway genes by RT-PCR (a–c) and quantified the results (d–f). To figure out the effect of Wnt signal in neural differentiation of stem cell, RT-PCR study was performed using primers of Wnt signal-related genes. (a and d) Most Wnt genes are overexpressed in primary hADSCs whereas the expression of Wnt5a is increased in NI-hADSCs. (b and e) Except Fzd3 and Fzd5, all Wnt receptor and coreceptor genes that are expressed in hADSCs are suppressed in NI-hADSCs. The expression of Fzd3 and Fzd5 is shown in NI-hADSCs. (c and f) The expression of Dvl1 and RYK is decreased in NI-hADSCs. Also, there is no change in the expression of either GSK3*β* protein or *β*-catenin. GAPDH was used as a control. The RT-PCR assay was repeated five times independently of different cells and the representative data are shown. The intensity of each gene was normalized to GAPDH. ^*∗*^ *P* < 0.05, ^*∗∗*^ *P* < 0.01 compared with the primary hADSCs. C, primary hADSCs; NI, NI-hADSCs.

To identify Wnt pathway genes at the molecular level, we performed RT-PCR analysis of Wnt pathway components including 4 Wnt families, 5 Wnt receptors, and 2 Wnt coreceptors. Following our previous study [9], hADSCs, which were already isolated from human fat tissue and characterized as MSCs, were differentiated into NI-hADSCs. Wnt2, Wnt4, and Wnt11 gene expressions were decreased whereas the expression of only Wnt5a gene was increased in NI-hADSCs (Figure 1(a)). Wnt receptors (Fzd2, Fzd4, and Fzd6) and coreceptors (LRP5 and LRP6) were all downregulated in NI-hADSCs, except Fzd3 and Fzd5, which were not expressed in hADSCs (Figure 1(b)). In addition, the expressions of RYK, which is a Wnt ligand receptor that can bind to the Wnt ligand, and Dvl1 were decreased in NI-hADSCs. However, GSK3*β* and *β*-catenin, which are the target genes for Wnt signalling, levels were not changed after neural differentiation (Figure 1(c)).

**Figure 2:**
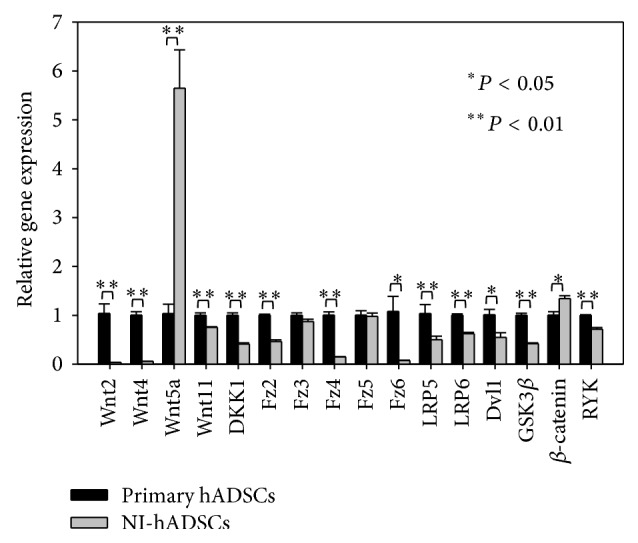
Real time RT-PCR analysis of Wnt pathway-related genes. Real time RT-PCR was performed to confirm the RT-PCR assay. The expressions of Wnt5a and *β*-catenin are increased and others are decreased in NI-hADSCs, significantly. Fz3 and Fz5 expressions are not changed. The real time RT-PCR assay was repeated four times independently of different cells and the representative data are shown. The intensity of each gene was normalized to GAPDH. ^*∗*^ *P* < 0.05, ^*∗∗*^ *P* < 0.01 compared with the primary hADSCs.

To confirm the expression of Wnt-related genes, we tested and analyzed Wnt genes and receptor related genes by real time RT-PCR. Interestingly, most of Wnt-related gene expressions were decreased but the expression of Wnt5a was highly increased after neural differentiation in hADSCs, significantly (Figure 2).

### 3.2. Wnt Pathway in NI-hADSCs

**Figure 3:**
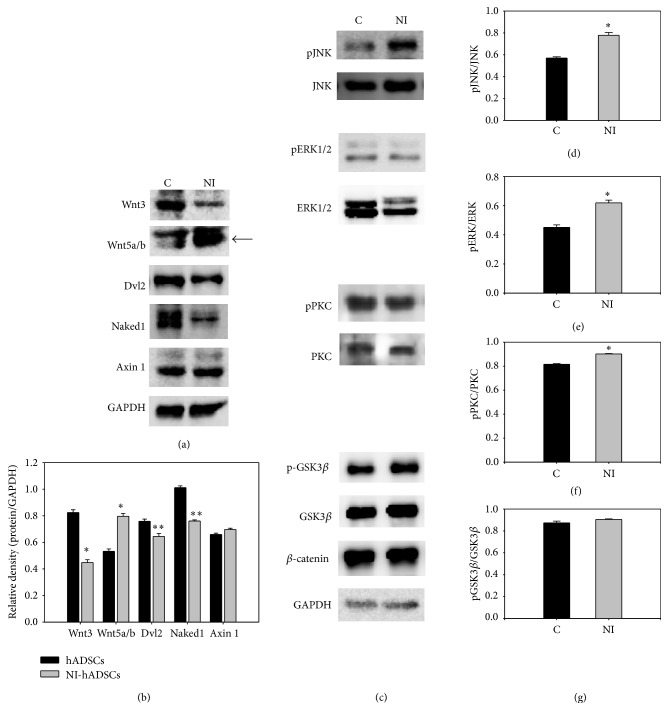
Expression of Wnt signal-related proteins. Western blot analysis demonstrated the Wnt signal in NI-hADSCs (a and b). The expression of Wnt3 is decreased in NI-hADSCs, but interestingly Wnt5a/b expression is upregulated after neural differentiation compared with primary hADSCs. Dvl2 protein is very slightly changed between primary hADSCs and NI-hADSCs. However, the expression of Naked1 is downregulated in NI-hADSCs compared with primary hADSCs. (c–g) Western blot analysis for Wnt signal-related proteins. The expression of phosphor-JNK, which is one of the downstream noncanonical pathways, is upregulated in NI-hADSCs compared with primary hADSCs. Also, ERK1/2 protein level is decreased after neural differentiation. (d–g) Quantified results of protein expression. The western blot was repeated three times independently of different cells and the representative data are shown. The intensity of each gene was normalized to GAPDH or *β*-catenin. ^*∗*^ *P* < 0.05 compared with the primary hADSCs.

We examined whether Wnts could be involved in promoting the neural differentiation. For analysis of Wnt signal pathway, we studied the Wnt-related pathway using western blot analysis.  Figure 3 showed that Wnt3 expression was decreased in NI-hADSCs compared with primary hADSCs. Importantly, Wnt5a/b protein was highly expressed in NI-hADSCs and those are consistent with RT-PCR results. So, we focused on the Wnt5a, one of the noncanonical Wnt molecules which are known to be expressed in proliferating cells and to increase during differentiation [15, 25]. Noncanonical pathway does not necessarily bind LRP5/6 complex, so we did not study LRP expression. Dvl2 and Naked1 protein levels were downregulated after neural differentiation (Figures 3(a) and 3(b)). The expression of Axin 1 was not changed in both primary hADSCs and NI-hADSCs.

### 3.3. Signalling Pathway in NI-hADSCs

**Figure 4:**
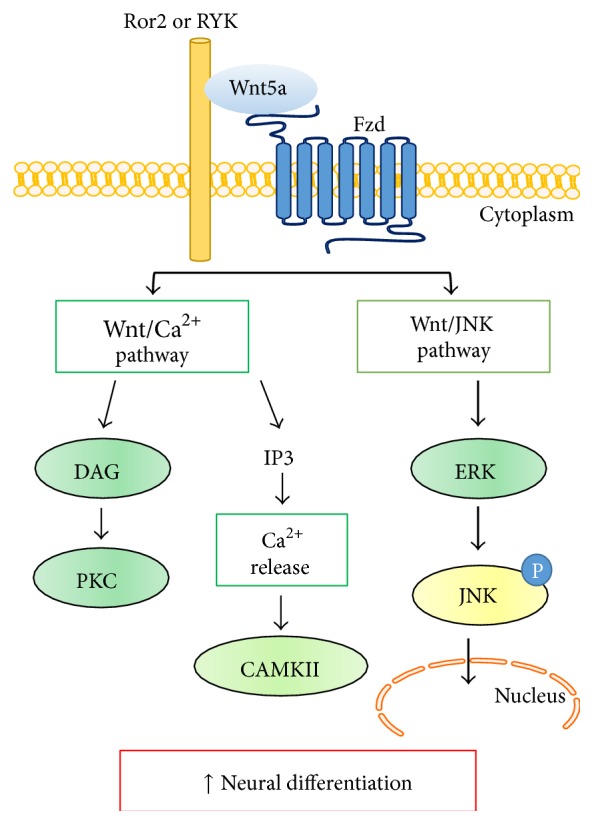
Noncanonical Wnt5a signalling: a simplified view. Wnt5a ligand binds to one of the seven transmembrane receptors of the Fzd family and results in Wnt/Ca^2+^ pathway or Wnt/JNK pathway activities. Previously, Wnt/Ca^2+^ pathway was already studied for other groups [27, 28]. Here, we demonstrate that Wnt5a upregulates ERK and phosphor-JNK signalling and activates the neural differentiation of hADSCs.

Noncanonical Wnt signalling has been shown in two different ways such as increases in intracellular Ca^2+^ and protein kinase C (PKC) level and an activation of c-Jun N-terminal kinase (JNK). To investigate the signalling pathways by which Wnt5a influences neural differentiation, we employed phosphor-specific antibodies to identify the signalling effectors activated by Wnt5a in NI-hADSCs. Western blotting was carried out to show expression of JNK and phosphor-JNK, key molecules of the noncanonical Wnt signalling pathway. The phosphor-JNK expression level was upregulated in relation to control GAPDH in NI-hADSCs compared with primary hADSCs (Figure 3(c)). Furthermore, extracellular signal-related kinase (ERK1/2) protein level was observed in primary hADSCs and downregulated in NI-hADSCs and phosphor-EKR1/2 level was not changed after neural differentiation. However, the GSK3*β* and phosphor-GSK3*β* protein levels were not changed in both the primary hADSCs and NI-hADSCs. The quantification data showed that the phosphor-JNK, phosphor-ERK1/2, and phosphor-PKC levels were increased in relation to total-JNK, total-ERK1/2, and total-PKC in NI-hADSCs, respectively (Figures 3(d)–3(g)). These data indicate that the JNK expression is the important modulator in hADSC during neural differentiation (Figure 4).

## 4. Discussion

In the last few years there have been wide interests in the role of Wnt signalling and it is also thought to play a key role in controlling stem cell fate. Thus, understanding the mechanisms that regulate Wnt signalling is of critical importance, especially in stem cell differentiation. In particular, Wnt5a/receptor tyrosine kinase-like orphan receptor 2 pathway regulated interleukin-1*β* or anti-tumor necrosis factor-*α* induced differentiation of human MSCs into osteoblasts [12, 13]. In addition, Wnt5a is known to be a mediator of axonal branching and growth in developing sympathetic neurons [26] and a promoter with JNK signalling of postsynaptic density in hippocampal neurons [17]. Here we aimed to study the expression of Wnt5a, which is a one of noncanonical Wnts, in neural differentiation of stem cells and to investigate further the role of Wnt signalling and JNK pathway. We found that the Wnt5a gene was mainly expressed in NI-hADSCs and upregulated the Fzd3 and Fzd5. Inestrosa's group reported that normally Wnt5a was expressed early in development and stimulated dendrite spine morphogenesis in the hippocampus, where it played a trophic role in neuronal differentiation and modulation of synaptic activity [29].

We observed that Wnt5a upregulated the Fzd3 and Fzd5 expressions and promoted the neural differentiation of hADSCs (Figure 1), showing constant high levels followed by real time RT-PCR (Figure 2). This expression pattern suggests that this ligand may have a role during neural differentiation. Yu et al. reported that Wnt3a and Wnt5a increased neuronal differentiation of neural progenitor cells [15]. Interestingly, our results showed that only Wnt5a was upregulated after neural differentiation and Wnt3 expression level was significantly decreased (Figures 1 and 2). It means that noncanonical Wnt pathway, especially Wnt5a, promotes neural differentiation of ADSCs. Additionally, we have evaluated the protein levels of GSK3*β* and *β*-catenin and found that protein levels were not changed after neural induction of hADSCs. These results depict the fact that the activation of the Wnt/*β*-catenin signalling pathway, which is a canonical Wnt pathway, is not involved in neural differentiation in our study.

There are two pathways of noncanonical Wnt signalling, Wnt/Ca^2+^, which downregulate PKC or calcium/calmodulin-dependent kinase II, or Wnt/JNK pathway by stimulated Wnt5a ligand [17, 30]. Zhang et al. reported that Wnt5a induced PKC activation and promoted axon differentiation in cultured hippocampal neurons [31]. These evidences demonstrate the Wnt pathways participation in the initial neural differentiation [21]. Interestingly, we also have observed the expression of Wnt5a and phosphorylation/activation of PKC after neural differentiation. In our study, we have shown that Wnt5a-mediated JNK activation is important in neural differentiation of hADSCs (Figure 3). This Wnt5a-mediated JNK activation is regulated under the control of not only Fzds but also ERK1/2 signalling (Figure 4).

In this study, we have focused on the stem cell differentiation, especially neural differentiation, through the noncanonical Wnt pathway. We have proved that the expression of Wnt5a, which is one of the noncanonical Wnts, gene level was increased after neuronal differentiation in hADSCs and protein level of downstream phosphor-JNK was also increased in the same way. Taken together, these data demonstrate that noncanonical Wnt signalling could activate the neural differentiation in hADSCs through the regulation of Wnt5a and the activation/phosphorylation of JNK pathway.

## 5. Conclusions

Wnt5a promotes noncanonical Wnt signalling followed by Fzd3 and Fzd5 expressions when primary hADSCs are differentiated into neural hADSCs* in vitro*. Future study will contribute to understanding the control of neural differentiation using Wnt signalling.

## Figures and Tables

**Table 1:** Sequence of PCR primers.

Gene	Sense	Antisense
Wnt2	TGGTGGTACATGAGAGCTACAGGTG	CCCCATTGTACTTCCTCCAGAGATA
Wnt4	GAGGAGACGTGCGAGAAACTCAA	ATCCTGACCACTGGAAGCCCTGT
Wnt5a	TTTTTCTCCTTCGCCCAGGTTGT	GGCTCATGGCGTTCACCAC
Wnt11	ACAACCTCAGCTACGGGCTCCT	CCCACCTTCTCATTCTTCATGC
Fzd2	CCATCCTATCTCAGCTACAAGTTTCT	GCAGCCCTCCTTCTTGGTG
Fzd3	TCCCCTCTGCCTGTATGTGGTAGT	GCTGCTCACTTTGCTGTGGA
Fzd4	CTCGGCTACAACGTGACCAAGAT	AATATGATGGGGCGCTCAGGGTA
Fzd5	TGCTACCAGCCGTCCTTCAGT	CCATGCCGAAGAAGTAGACCAG
Fzd6	TTTTGTCTTTGTGCAACTCTGTTCA	AGCCAATTCTGGTCGAGCTTTTG
LRP5	TCATTGTGGACTCGGACATTTACTGG	GTAGAAAGGCTCGCTTGGGGACAG
LRP6	CATTGTCCCAGAGGCTTTCCTTTTG	TAGGTTTTCCACCCCATTCAGTCCA
Dvl1	CCGCTGACGGTGAAGAGTGACAT	CCTGACTGCGTGGGCTGCTG
RYK	ATTTCCTGCACTTCACCTGG	CTTTGGCCTCCAAAAGAGTG
GSK3*β*	CAGCAGCCTTCAGCTTTTGG	CCGGAACATTGTCCAGCACCAG
*β*-catenin	GCTGATTTGATGGAGTTGGACATGG	GCCAAACGCTGGACATTAGTGG
GAPDH	CATGACCACAGTCCATGCCATCACT	TGAGGTCCACCACCTGTTGCTGTA
